# Progress in Surgery

**Published:** 1900-07-14

**Authors:** 


### Progress in Surgery.

### HERNIA.

(*Continued from page* 240)

**Encysted Hernia.**—This is the subject discussed by Moynihan in his second Arris and Gale lecture.[4] Encysted hernia is defined as that form of hernia in which the sac, with its contents, wholly covered on the outer side by a reflected layer of serous membrane, hangs downwards from the internal ring into the cavity of the processus vaginalis. The tunica vaginalis may or may not be shut off from the processus vaginalis. Moynihan remarked that "until Lockwood carefully defined and restricted the term 'infantile,' many ruptures of this infantile form were certainly looked upon as 'encysted,' and to some extent the error still persists." Lockwood applies the word infantile to that variety of hernia in which a separate peritoneal pouch is dragged down into the scrotum, behind the tunica vaginalis, probably by an aberrant attachment of the gubernaculum. Three forms of encysted hernia were described by the lecturer: (1) Encysted hernia of the tunica vaginalis, the testicle lying at the bottom of the cavity; (2) encysted hernia of the tunica vaginalis, the testis lying at the lower end and "apex" of the encysted mass; and (3) encysted hernia of the funicular process. In the first variety, on opening what appears to be the sac, a little fluid, as a rule, escapes. It is then found that the sac has not been opened, but instead a serous cavity—the tunica vaginalis—with the testicle lying at the bottom. Pendent from the neck, which is at or nearly at the internal ring, is a thick elongated mass covered by a reflection of the tunica vaginalis aptly compared to the "teat of a cow" (Bourguet). This is the hernial sac, and to open it three layers of peritoneum have to be divided. Moynihan believes these hernias arise as Bourguet first suggested. According to this theory the funicular process closes only at one spot near the internal ring. A hernial sac now forms in the usual manner from the parietal peritoneum, originated possibly by a small dimple at the point of closure of the funicular process. This "fusion" point will then form the tip of the advancing hernial mass, which invaginates the peritoneum of the enclosed portion of the funicular process. Cases have been recorded in which the apex of the hernial sac has ruptured, and the bowel, escaping into the tunica vaginalis, has been strangled at the point of rupture. A hernia has also been described which was encysted in a hæmatocele of the tunica vaginalis. In the second variety of encysted hernia described, "the testis, instead of lying on the floor of the tunica vaginalis, lies at the apex of the encysted tumour, at the orifice, so to speak, of the teat" (Moynihan). In dealing with these cases by operation, it is not recommended that a new and smaller tunica vaginalis for the testicle be made. When this has been done it has led to the occurrence of hydrocele.

**Sliding Hernias of the Cæcum and Sigmoid Flexure.**—Robert F. Weir[5] has written a paper on this subject, from which the following remarks have been abstracted: The sac of these hernias is imperfect, for a portion of the protruded bowel is still outside the peritoneum, and has escaped from the abdomen without pushing before it the parietal layer of the peritoneum. In the congenital forms the descent of the bowel is produced by the agency of the gubernaculum testis. On the other hand, most of the hernias of the cæcum and sigmoid are provided with a complete sac, owing to the fact that normally these structures are generally completely covered by peritoneum. When the colon has a long mesentery, the cæcum may appear in a left-sided hernia, and the sigmoid in a right-sided one. Macready says that in 57 instances of cæcal hernia, five were right femoral, and one left femoral. Hernias spoken of as those of the slipped cæcum and sigmoid really are hernias involving the ascending or descending colon. They present difficulties of operative reduction which are not often satisfactorily overcome. These hernias *par glissement* are generally so irreducible that special surgical interference is demanded. If they become strangulated, which is possible, but rare, the surgeon may have to relieve the constriction only, and forego reduction and radical cure. It is usually the postero lateral aspect of the bowel which is uncovered by the peritoneum, and it is the connective tissue situated there, often dense and thickened, which holds down the bowel and prevents its easy reduction. It is difficult to determine the nature of these ruptures before operation. They are only reducible in their early stages. When irreducible they are usually scrotal. Sometimes when left-sided their existence may be suspected because there is inability to inject more than a small quantity of fluid per rectum, or because such an injection distends the bowel in the scrotum appreciably. The associated hernial sac in front often contains small intestine or omentum. The uncovered portion of bowel is apt not to be recognised, and so accidentally wounded in an operation, owing to the absence of its tell-tale peritoneal covering. Very little information as to the treatment of these herniæ is to be found either in text-books or special works on hernia. Ladroitte, in 1882, stated that in a cadaver he could only bring about reduction by completely separating the bowel along its cellular attachment up to the external ring. In practice the majority of cases have been unsatisfactorily treated, vain attempts having been made to push up the attached bowel toward the internal ring, and hold it there by sutures passed through the canal below it. Separation of the bowel from its subperitoneal bed may be difficult owing to the deneness of the tissue, and the nutrient vessels of the intestines may be so damaged as to bring about its gangrene. Froelich's (of Nancy) collection of 21 instances of these hernias is quoted in illustration of the risk of separating the bowel where adherent. Many of these cases remained uncured after the operation; in three fæcal fistulæ resulted. Juillard once resorted successfully to the severe measure of cutting away the protruding bowel and joining the divided intestines with a Murphy's button. Weir, in two cases of cæcal hernia of this kind, resorted to the expedient of covering the raw surface of the intestine, made by detaching it, with peritoneal flaps derived from the sac. The sac was dissected up on each side of the bowel by incisions parallel to its long axis, beginning at the top on a level with the internal ring, and ending at the bottom a short distance below the bowel, these lower extremities being joined by a transverse incision. Having then separated the bowel, the loosened peritoneum is turned backward and sutured behind the gut as far as practicable. A radical cure is then completed, preferably with the removal of the cord and testicle of the affected side.

[4] Op. cit. [5] Med. Rec., Feb, 1900.

